# Short- and Long-Term Outcomes of Pancreatic ERCP in Pre-Teens: A Swedish Single Center Study

**DOI:** 10.3390/jcm15103941

**Published:** 2026-05-20

**Authors:** Chiara Maria Scandavini, Alexander Waldthaler, Roberto Valente, Mari Hult, Fredrik Lindgren, Johannes Matthias Löhr, Asif Halimi, Ernesto Sparrelid, Urban Arnelo

**Affiliations:** 1 Department of Diagnostics and Intervention, Surgery, Umeå University, 901 87 Umeå, Sweden; chiara.scandavini@regionvasterbotten.se (C.M.S.); roberto.valente@umu.se (R.V.); asif.halimi@regionvasterbotten.se (A.H.); 2 Department of Upper Abdominal Diseases, Karolinska University Hospital, 171 76 Stockholm, Sweden; alexander.waldthaler@ki.se (A.W.); mari.hult@regionstockholm.se (M.H.); matthias.lohr@ki.se (J.M.L.); ernesto.sparrelid@ki.se (E.S.); 3 Department of Medicine Huddinge (MED H), Karolinska Institutet, 141 83 Stockholm, Sweden; 4 Department of Surgery, Division of Surgical Oncology, University of Colorado School of Medicine, Aurora, CO 80045, USA; 5 Division of Surgery and Oncology, CLINTEC, Karolinska Institutet, 141 86 Stockholm, Sweden; fredrik.lindgren@regionstockholm.se; 6 Division of Pediatric Gastroenterology, Hepatology and Nutrition, Karolinska University Hospital, 171 76 Stockholm, Sweden

**Keywords:** hereditary pancreatitis, pediatric ERCP, post-ERCP complications, pancreatic stenting, pediatric endoscopy

## Abstract

**Background/Objectives:** Endoscopic retrograde cholangiopancreatography (ERCP) in children with acute or chronic pancreatitis, or following pancreatic trauma, is technically demanding and may be associated with an increased risk of complications. Evidence on technical success and complication rates in preadolescent children is limited. This study aimed to evaluate the short- and long-term outcomes of ERCP with pancreatic stenting in children with pancreatic conditions. **Methods:** In this retrospective single-center cohort study, consecutive patients aged ≤12 years who underwent ERCP with pancreatic stenting for acute or chronic pancreatic diseases or pancreatic trauma were included. Demographic, clinical, and procedural data were collected, and complications and clinical response, were assessed. **Results:** A total of 20 patients (mean age 7 years, range 2–12; 45% female) underwent 62 ERCP procedures for pancreatic indications. Nine patients (45%) had a known genetic mutation. Post-ERCP pancreatitis occurred in 2 procedures (3.2%), and bleeding in 1 procedure (1.6%). No perforations or procedure-related mortality were observed. Technical success was achieved in 57/62 procedures (91.9%), with associated improvement in symptoms, pain, or inflammatory markers. **Conclusions:** In this pilot study from Sweden, ERCP with pancreatic stenting appears to be a feasible therapeutic option in pre-teen children with pancreatic diseases, with a good technical success rate and relatively low complication rates. Further studies are warranted to better define long-term outcomes in this population.

## 1. Introduction

Endoscopic retrograde cholangiopancreatography (ERCP) is a technically challenging procedure that carries up to an 8–15% risk of developing post-ERCP pancreatitis (PEP) [1,2]. Known risk factors for PEP include patient-related factors such as young age, female sex, a history of prior acute pancreatitis (AP), and the absence of chronic pancreatitis (CP). Procedure-related factors are also significant and are primarily associated with pancreatic ERCP procedures, particularly when specific interventions are undertaken, such as pancreatic sphincterotomy, contrast medium injection, or the placement of multiple stents within the main pancreatic duct [1,3].

A history of acute pancreatitis and recurrent acute pancreatitis (RAP) represents a significant patient-related risk factor for post-ERCP pancreatitis (PEP), as individuals with previous episodes of acute pancreatitis may exhibit increased pancreatic susceptibility to inflammatory stimuli due to prior parenchymal injury and altered ductal physiology [4]. Repeated inflammatory insults may result in impaired pancreatic juice outflow and increased acinar cell sensitivity, rendering the pancreas more vulnerable to even minor procedural trauma or transient elevations in ductal pressure during ERCP, thereby triggering a renewed inflammatory cascade [5]. A highly functional pancreatic parenchyma, together with a small-caliber bile duct and main pancreatic duct, may also constitute potential risk factors. From this perspective, the pediatric population may present additional disease-specific predispositions to the development of post-ERCP pancreatitis [1].

RAP and CP in children differ etiologically from their adult counterparts; nevertheless, pediatric patients may still benefit from interventions aimed at decompressing the ductal system, alleviating symptoms, and preserving pancreatic function. In this context, ERCP, owing to its minimally invasive nature compared with surgical approaches, may be considered a first-line therapeutic option [6]. Endoscopic treatment can offer significant benefits in pediatric patients, with good safety and efficacy profiles in reducing pain and improving pancreatic drainage [7,8]. In particular, pancreatic stent placement has proven to be a useful strategy in both CP and RAP, contributing to the reduction of painful episodes and relapses [9].

On the other hand, pancreatic ERCP in pediatric populations might pose additional challenges, considering that the main pancreatic duct diameter in healthy children has a mean value of approximately 1.6 mm, with upper limits reaching only about 1.9 mm in children up to 10 years of age [10,11,12].

Although various studies have shown the technical feasibility and safety of ERCP in children for biliary and mixed biliary/pancreatic indications [13,14], pediatric patients with acute or chronic pancreatitis or pancreatic trauma may be at higher risk for post-ERCP complications compared to their adult counterparts. In addition, there are only a couple of previous studies that have reported outcomes of ERCP in pediatric patients with exclusively pancreatic indications [6,7]. In contrast, most previous studies have typically included heterogeneous cohorts with biliary and/or pancreatic indications and have predominantly involved adolescent patients, with ages extending up to 18.4 years [15]. Data on ERCP outcomes specifically in pre-teens undergoing the procedure for primary pancreatic indications remain underrepresented in the literature [6,16]. Much of the existing evidence comes from multicenter studies or registry-based research [17,18].

The aim of the present study is to evaluate both the short- and long-term outcomes of ERCP in a preadolescent pediatric population at a tertiary-level Swedish reference center.

The primary endpoints of the study were the evaluation of the effectiveness of endoscopic treatment (resolution of the acute inflammatory condition and/or improvement in pain) and the assessment of post-procedural complications in children up to 12 years of age. Secondary outcomes were the type of stenting used and technical aspects such as the need for pre-cut sphincterotomy/pancreatic sphincterotomy, the use of dilation of the main pancreatic duct in the same population.

## 2. Materials and Methods

### 2.1. Population

We performed a single-center retrospective cohort study at Karolinska University Hospital, Stockholm, Sweden. The study included pediatric patients aged ≤12 years who underwent ERCP for acute or chronic pancreatic diseases between September 2003 and June 2019. All ERCP procedures were performed by a single experienced endoscopist (UA).

Patients older than 12 years or those undergoing ERCP for non-pancreatic indications were excluded. Eligible patients were identified through the hospital’s endoscopy database, and clinical data were retrieved from electronic medical records.

The diagnosis of acute pancreatitis was established according to the revised Atlanta classification [14], requiring at least two of the following three criteria: (1) abdominal pain consistent with pancreatitis, (2) serum amylase and/or lipase levels at least three times above the upper normal limit, and (3) characteristic findings on contrast-enhanced computed tomography (CECT) or magnetic resonance imaging (MRI).

Chronic pancreatitis was diagnosed based on a combination of clinical and radiological findings, including persistent or recurrent abdominal pain, loco-regional complications, and imaging evidence of pancreatic ductal abnormalities (e.g., strictures, dilation, or intraductal stones) or parenchymal changes [19].

In patients with idiopathic pancreatitis, genetic testing was performed using a standardized panel to identify mutations associated with pancreatic disease. The panel included, but was not limited to, CPA1, CTRC, SPINK1, PRSS1, and CFTR genes. Genetic analyses were conducted according to institutional protocols [20].

For each ERCP procedure, detailed technical and clinical data were collected, including indication for ERCP, cannulation technique, use of minor papilla access, presence of anatomical abnormalities (e.g., *pancreas divisum*), pancreatic duct features (strictures, dilation, stones), type, size, and number of stents placed, and use of mechanical dilation. Multiple stenting with or without dilation was applied for the management of main pancreatic duct (MPD) strictures. Patients were followed through medical records to assess both short- and long-term outcomes. Outcomes included procedure-related complications (e.g., bleeding, perforation), recurrence of pancreatitis, and mortality. Clinical success of ERCP was defined as either complete resolution of pain or a clinically meaningful improvement in symptoms sufficient to permit a stable transition into adulthood, or to allow recovery from the acute condition to the extent that subsequent elective surgery could be safely undertaken. In the assessment of long-term outcomes, the need for surgical intervention was considered a long-term failure of endoscopic treatment when it was related to the management of pain or to a condition not amenable to endoscopic resolution. In contrast, surgery indicated an underlying oncologic disease was not regarded as a failure of endoscopic therapy.

### 2.2. Endoscopic Technique

In patients with chronic pancreatitis presenting with a pancreatic duct stricture (with or without intraductal stones), the initial ERCP was primarily focused on traversing the stricture. After successful cannulation, a guidewire was advanced across the stenosis, followed by dilation and placement of at least one pancreatic stent, even if of small caliber (4–7 Fr). Stone extraction at this stage was considered secondary and performed only if readily achievable; otherwise, it was deferred until after adequate stricture dilation. A second ERCP was performed after 3–4 months. During this session, previously placed stent(s) were removed, the stricture was re-dilated (most commonly using a 4 mm balloon), and the stenting strategy was aimed at increasing the overall drainage diameter. This was achieved either by placing a larger-caliber stent or by inserting an additional stent of the same caliber. At each stent exchange, gentle ductal downstream irrigation and clearance was carried out using a retrieval balloon (8–9 mm), starting near the papilla and progressively advancing deeper into the main pancreatic duct. Subsequent stent exchanges were performed at 3–6 month intervals, following the same principles of repeated dilation, ductal clearance, and progressive stent upsizing. This stepwise approach was continued until minimum three stents of 8.5 Fr were in place. The final goal was to maintain at least three 8.5–10 Fr stents in situ for approximately 12 months. At the end of this period, all stents were removed and the pancreatic duct was cleared from debris, as described above.

All procedures were performed under general anesthesia with orotracheal intubation in an inpatient setting. Adult therapeutic duodenoscopes (Olympus Corporation, Tokyo, Japan) were used in most cases, while a pediatric duodenoscope (Olympus PJF-160, outer diameter 7 mm) was used in children younger than 4 years. When performing endoscopic retrograde cholangiopancreatography (ERCP) in very young children—particularly those under four years of age—it was sometimes necessary to use ultra–slim accessories, including narrow-caliber catheters and sphincterotomes. For example, a tapered catheter such as the ContourTM ERCP cannulas 5-4-3 (Boston Scientific, Marlborough, MA, USA; 3 Fr in the distal tip) may be employed, along with a sphincterotome (DASH-21, 5.4 Fr Wilson-Cook, Bloomington, IN, USA) featuring a short cutting wire of approximately 15 mm. The use of a short cutting wire was essential in very small children due to the limited luminal diameter; standard sphincterotomes are often unsuitable in this setting, as the cutting wire may remain partially within the instrument rather than being adequately exposed. In the smallest children, it was sometimes also necessary to use guidewires with much smaller diameter, guidewires as small as 0.021–0.018 inch, or even smaller.

### 2.3. Statistical Analysis

Continuous variables were analyzed using one-sample Student’s t-test and presented as mean value with 95% CI. A *p*-value < 0.05 was considered statistically significant. Statistical analysis was performed through MedCalc, vers 23.4.8 MarienKirke, Schaerbeek, Belgium.

The study was approved by the Swedish Ethical Review Authority (Dnr 2019-05937) and conducted in accordance with the Declaration of Helsinki.

## 3. Results

### 3.1. Indications for ERCP

Twenty patients underwent 62 ERCP procedures for primary pancreatic indication (acute pancreatitis, chronic pancreatitis, pancreatic trauma). The mean age was 7.6 years (95% CI: 6.7–8.4). Median follow-up was 68 months (95% CI: 46–108 months). Nine children (45.0%) were female. There were six children (30.0%) with pancreatic trauma. Overall, nine patients (45.0%) had a known genetic mutation associated with either an increased risk of chronic pancreatitis or an increased lifetime risk of cancer: one patient (5.0%) had a CPA1 mutation, one patient (5.0%) had a CTRC mutation, two patients (10.0%) had a SPINK1 mutation, one patient (5.0%) had a MEN1 mutation, three patients (15.0%) had a PRSS1 mutation, and one patient (5.0%) had a homozygous MUT C655A mutation [21].

In total, 34 procedures (54.8%) were performed because of chronic pancreatitis, six (9.6%) because of acute pancreatitis, 10 (16.1%) because of a main pancreatic duct rupture, and 11 (17.7%) because of recurrent acute pancreatitis. A total of four children had *pancreas divisum*, representing 25 procedures (40.3%). Patient characteristics and procedural characteristics are summarized in Table 1a,b.

In addition to the 62 ERCPs, a simultaneous EUS was performed in eight (12.9%) procedures and a pancreatoscopy in two (3.2%) procedures.

### 3.2. Endoscopic Treatment

Minor papilla cannulation was performed in 10 procedures (16.1%), and pancreatic sphincterotomy in 15 procedures (24.2%). Intraoperative pancreatography showed a dilated (4–11 mm) main pancreatic duct in 48 procedures (77.4%), strictures in 36 procedures (58.0%), stones in 22 procedures (35.4%), plugs in 26 procedures (41.9%), and pseudocysts in 25 procedures (43.1%). Balloon dilation was performed in 15 procedures (24.1%). Catheter-assisted dilation was performed in two procedures (3.2%). Pancreatic stenting was performed in 54 ERCP procedures (87.0%), with 38 procedures (61.2%) performed following a previously positioned stent. Table 2 summarizes the characteristics of endoscopic and stent treatment.

### 3.3. Outcomes of Endoscopic Treatment

Overall, 91.9% of the procedures were technical successful. In one procedure (1.6%), performed on an 11-year-old boy with a CTRC mutation, jaundice, and focal pancreatitis, the operator failed to achieve deep cannulation of the main pancreatic duct. No patient mortality was recorded as a result of ERCP related complications. Mild post-ERCP acute pancreatitis occurred after two of the procedures (3.2%). After one procedure (1.6%) bleeding occurred, but it resolved spontaneously and the patient did not require blood transfusion. No procedure-related perforation occurred. In another ERCP (1.6%), performed on an 11-year-old boy, who was conservatively treated for a main pancreatic duct rupture with pseudocyst formation, the operator failed to bridge the rupture/pseudocyst. Nevertheless, the patient was successfully treated with a combination of a trans-papillary pancreatic stent and an endoscopic ultrasound-guided transgastric double-pigtail stent. The patient developed a chronic pancreatic fistula between the distal pancreas and the stomach, with the head of the pancreas draining into the duodenum. He remained asymptomatic and had normal growth and maturation into adulthood.

Despite endoscopic treatment, one patient experienced recurrent episodes of acute pancreatitis during long-term follow-up and eventually underwent elective surgery. Another patient developed chronic pain and eventually underwent total pancreatectomy with autologous islet cell transplantation. In two patients (10.0%) with main pancreatic duct rupture, bridging the defect was not possible, due to long dehiscence. Both patients first received downstream pancreatic stents and subsequently were treated with spleen-preserving distal pancreatectomy. In light of the absence of data on pain scales, more recent biochemical and radiological parameters, and a more comprehensive assessment of procedural success, we considered that technical success was achieved in 91.9% of cases, whereas clinical success was observed in 80.0% of cases, taking into account that four out of 20 patients ultimately required surgical intervention. Technical success was assessed on a per-procedure basis, while clinical success was evaluated at the patient level based on outcomes. Table 3 summarizes the outcomes of endoscopic treatment.

In our series, 91.9% of procedures reached high rate of symptom control and effective long-term management of chronic pancreatitis. Table 4 provides a demographic overview and describes short- and long-term outcomes at patient level.

## 4. Discussion

ERCP is considered a challenging procedure when performed in pediatric patients. In the current study, we present the results of a single tertiary-level Swedish center series performed by one expert operator for the treatment of 20 children aged between 2 and 12 years of age with acute and chronic pancreatic diseases or pancreatic trauma. In our series 91.9% of the ERCP-procedures were technically successful, leading to either complete pain relief or a significant improvement in symptoms to allow for a stable transition into adulthood. When ERCP failed, either we managed to effectively treat the patient with EUS guided drainage in the acute phase, or we were able to perform pancreatic surgery later, when the child was grown-up and could better handle potential complications of pancreatic surgery.

To the best of our knowledge, there are no other studies in the literature reporting on a series of pure pancreatic ERCP procedures with pancreatic stenting in pre-teens.

A Meta-Analysis of 52 studies conducted on children for either biliary indications (48.0%) or mixed biliary/pancreatic indications (41.0%) has shown an overall complication risk of 7.0%, with a pooled incidence rate of post-ERCP pancreatitis like that observed in adults [13,22]. In our study, ERCP significantly impacted the outcomes of pediatric patients with hereditary chronic pancreatitis with an acceptable rate of post-ERCP pancreatitis, which is similar to the 3.2–5.4% reported in the adult Swedish population by the national quality register GallRiks [22]. Likewise, Shah et al. reported a 5.2% rates of post-ERCP pancreatitis in a large multi-institutional study of 110 pediatric patients [17].

However, the incidence of post-ERCP acute pancreatitis in patients undergoing ERCP for purely pancreatic indications may be higher. Recently, Garg L. et al. reported a series of 150 pediatric patients, 88 of whom were treated for pancreatic disorders, including 45 with pancreatic trauma, 21 with main pancreatic duct rupture, and 16 with recurrent pancreatitis. The authors reported a 94.3% success rate for ERCP and acceptable postoperative complication rates, with 10.0% of patients developing post-ERCP pancreatitis [23]. Similarly, Cheng C. et al. analyzed data from 245 patients under 17 years of age who underwent ERCP, 111 of whom had pancreatic disease and 93 had biliary pathology. The reported rate of post-ERCP pancreatitis was 9.7% [24].

Hassan M.A. et al. presented a large cohort from Cincinnati Children’s Hospital Medical Center, including 402 pediatric patients who underwent 736 ERCP procedures for pancreatic and biliary indications. A similarity with our series is the limited number of operators, which reduces the potential for heterogeneity due to variations in techniques and represents a bias in large multicenter or register studies. Another strength of their study is the large number of patients and procedures analyzed. The authors found no association between genetic mutations and post-ERCP pancreatitis, a finding that aligns with our results. However, the rate of post-ERCP pancreatitis was lower in our study compared to their study (3.2% vs. 12.8%).

Vegting I.L. et al. described a series of 61 younger patients who underwent ERCP. Their study included children aged between 3 days and 16.9 years, with 50.8% of patients being under one year old. However, unlike our series, only 16.3% of their patients had pancreatic indications. The authors reported pancreatic stent placement only in eight patients and pancreatic duct or papillary stricture dilation in two patients. Additionally, two patients were found to have *pancreas divisum*, while only one had hereditary pancreatitis [21]. In contrast, 45.0% of the patients in our series had genetic mutations.

The present study has several limitations, including its retrospective design, relatively small sample size, and potential heterogeneity in ERCP indications. The absence of a control group restricts the ability to draw definitive conclusions regarding comparative efficacy. Furthermore, potential selection bias and incomplete data may have influenced the results. Additionally, the lack of a standardized pain scale prevented an objective assessment of pain reduction.

An important methodological limitation of the current study is that it integrates procedure-level and patient-level analyses in a manner that complicates overall interpretation. Although the cohort comprises only 20 children, a total of 62 ERCP procedures are reported, including numerous repeat interventions. Short-term outcomes (e.g., post-ERCP pancreatitis, bleeding, and technical success) are analyzed on a per-procedure basis, whereas long-term outcomes (such as recurrent pancreatitis, need for surgery, and follow-up data) are presented also at the patient level. Given that multiple procedures were performed in the same individuals, these observations are not statistically independent; consequently, procedure-based estimates may underestimate the true patient-level burden and may convey a degree of precision not fully supported by the dataset.

The patient cohort is notably heterogeneous; however, the results are frequently interpreted as though all pancreatic indications were equivalent. The series encompasses children with chronic pancreatitis, acute pancreatitis, recurrent acute pancreatitis, pancreatic trauma, main pancreatic duct rupture, *pancreas divisum*, pseudocysts, ductal stones, strictures, and genetic forms of pancreatitis—conditions that represent distinct clinical entities with different pathophysiological mechanisms, procedural objectives, and anticipated outcomes. However, given the limited number of patients included, conducting a meaningful analysis of statistical significance across these subgroups is challenging, as the study would lack sufficient statistical power.

Despite these shortcomings, we believe our study provides meaningful insights into both the short- and long-term effects of ERCP, and into its potential role in supporting the transition of pediatric patients toward adulthood. In addition, the grown up patient is able to make autonomous decisions. Furthermore, our study focuses specifically on ERCP outcomes for pancreatic indications, with a highly homogeneous cohort in terms of age (limited to patients aged ≤12 years) and operator expertise thereby enhancing the internal validity of our findings.

**Figure 1:**
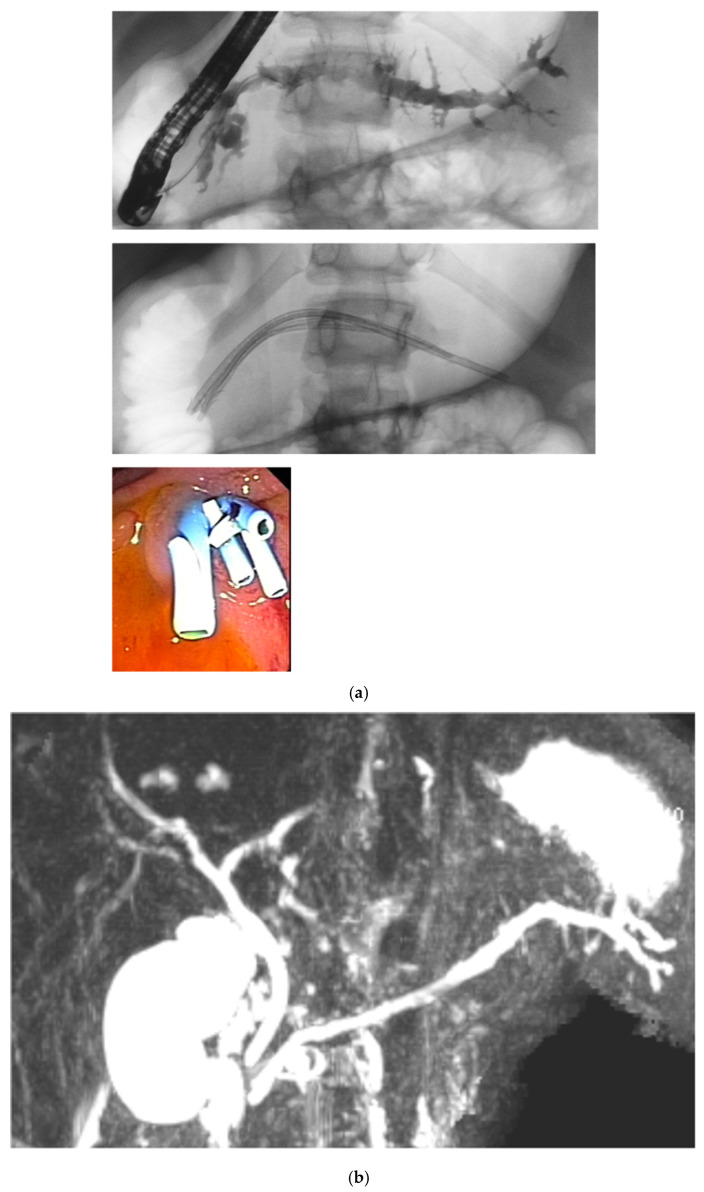
(**a**) An 11-year-old boy with severe chronic pancreatitis with multiple strictures due to SPINK1 mutation. He was treated with multiple stenting over three years. After his last ERCP and following stent removal, the patient did not require further hospitalization during his childhood. (**b**) Follow-up MRCP performed one year after stent removal in the same patient presented in the previous figure.

**Figure 2:**
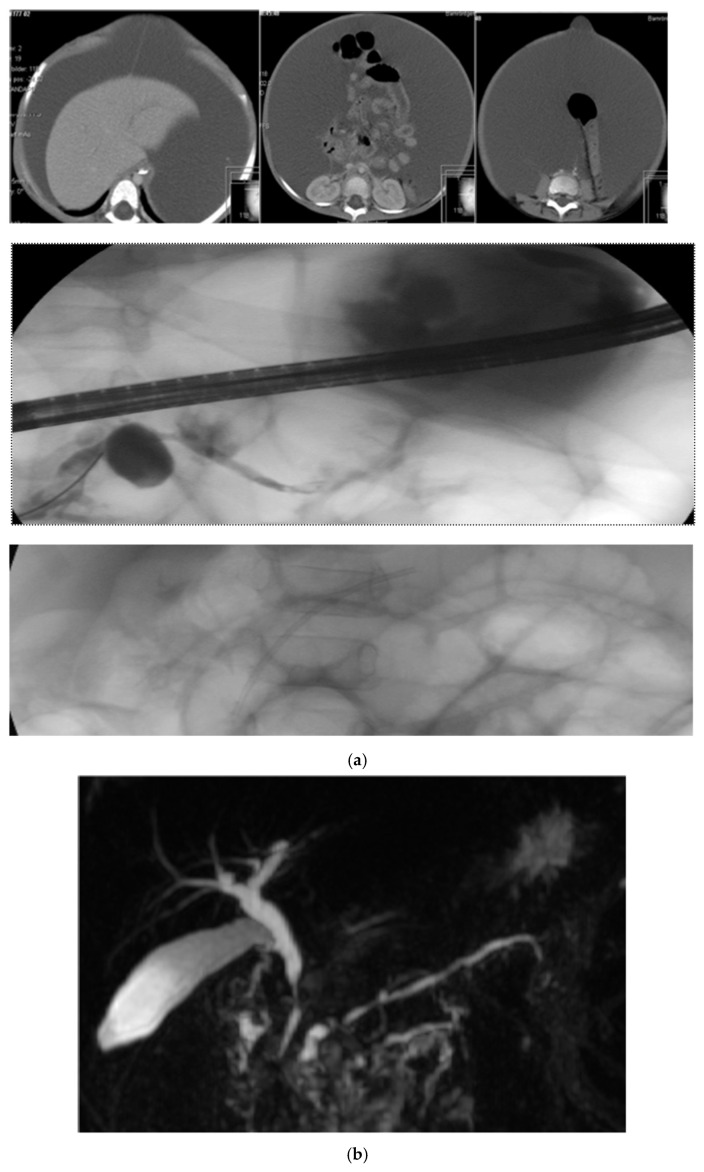
(**a**) A two-year-old girl with main pancreatic duct (MPD) rupture, pseudocyst formation, and massive pancreatic ascites secondary to hereditary pancreatitis (R122H mutation). She underwent a total of seven ERCP procedures between 2003 and 2008. Thereafter, she experienced a normal upbringing and development throughout the remainder of her childhood. (**b**) MRCP of the same girl eight years later.

A separate comment is warranted regarding the variant of pancreatic multistenting developed and used in the present study. Compared with the original technique described by Costamagna et al. for benign strictures of the biliary tract and later used in the pancreatic ducts [25,26,27,28]. The original technique for the pancreatic ducts implies dilation of a stricture according to the width of the pancreatic duct followed by deployment of the maximum number of stents allowed for 6–12 months. The variant of multiple stenting used in the present study likewise involves progressive stenting of the main pancreatic duct or the accessory pancreatic duct but represents a more gradual dilation of strictures and non-dilated segments downstream of stones and pseudocysts. This is achieved by focusing on passing the stone/stricture during the first ERCP session to decompress the upstream dilation mostly by insertion of a single 4 or 5 or 7 Fr plastic stent. This first intervention is then followed by repeated sessions of stent exchanges every three to four months. At each ERCP session, the diameter of the stents increase by inserting stents with larger diameter and/or increasing the number of stents. The final goal is to place at least three 8.5 Fr stents for 12 months, after which all stents are removed. At each ERCP, all stents are removed followed by careful and gentle, backward irrigation using a small balloon to clear the duct from debris. In our view, this technique allows for a more stepwise dilation of the stenotic segment and could minimize the pancreatic trauma, which in turn potentially could reduce the risk of post-ERCP pancreatitis. Indeed, in our series, the rate of post-ERCP complications was very low, despite the inclusion of high-risk patients undergoing high-risk interventions, Figure 1 and Figure 2.

Although this was not a primary objective of the study, the endoscopic technique evolved over time. In particular, placement of pancreatic stents was performed after irrigation of the endoscope working channel with 2–3 mL of 70% ethanol, with the aim of reducing the bacterial load and preventing early bacterial contamination of the stent, which might promote biofilm formation and consequently lead to earlier stent dysfunction. It should be emphasized that this technique was not subjected to quantitative evaluation, and further studies will be required to clarify its potential efficacy. Nevertheless, although not quantifiable, the endoscopic technique was progressively improved and refined over time, in accordance with the learning curve in pediatric endoscopy and the increasing operator experience.

In our opinion, endoscopic treatment, performed using the described step-up approach, is an effective option for managing acute and chronic inflammatory pancreatic conditions in pre-teens, particularly in the short term. The “gradual step-up approach” described in this study should be regarded primarily as a potential alternative therapeutic approach rather than a validated technique. Its interpretation must be approached with caution, particularly given that the present work is a small, uncontrolled, and descriptive series. Importantly, there was no predefined intention to systematically compare complication rates between different techniques.

Further studies, particularly multicenter randomized controlled trials, are needed to better define the indications and identify the patient populations that would benefit most from endoscopic treatment of acute and chronic pancreatitis.

## 5. Conclusions

In this Swedish single-center series, ERCP and pancreatic stenting in preadolescent children with pancreatic trauma, acute, or chronic pancreatitis seems to be feasible and safe, with low complication rates and favorable outcomes in terms of pain control and resolution of acute inflammation.

A key strength and innovative aspect of this study is the long-term, real-world evaluation of ERCP in a pediatric population managed by a single expert operator, providing procedural consistency and detailed characterization of pancreatic duct interventions, including the use of advanced techniques such as multiple stenting. Additionally, the integration of genetic testing in idiopathic cases offers further insight into the underlying etiology of pancreatic disease in this population.

In this study, we describe a variant of pancreatic multistenting. This approach is based on a more delicate and stepwise management of MPD strictures, aiming to achieve gradual ductal dilation while minimizing procedural trauma.

However, several limitations should be acknowledged. The retrospective design and single-center setting may limit the generalizability of the findings. The relatively small sample size and the absence of a control group restrict the ability to draw definitive conclusions regarding comparative efficacy. Furthermore, potential selection bias and incomplete data inherent to retrospective analyses may have influenced the results. Additionally, the lack of a standardized pain scale prevented an objective assessment of pain reduction.

Future prospective, multicenter studies with larger patient cohorts are needed to better define the role of ERCP in pediatric pancreatic diseases, particularly in preadolescent patients, to help bridge them to adulthood, when more invasive approaches can be performed without affecting development. Standardization of indications, techniques, and outcome measures, as well as longer follow-up, will be essential to establish evidence-based guidelines and optimize patient selection.

## Figures and Tables

**Table 1:** (**a**) Patient Characteristics. (**b**) Procedural Characteristics.

(**a**)	
Number of patients	20
Male sex	11/20 (55.0%)
Mean age, years (range)	7.6 (2–12)
Known genetic mutations CPA1 CTRC SPINK1 MEN1 PRSS1 C655A mutation (homozygous)	9/20 (45.0%) 1/20 (5.0%) 1/20 (5.0%) 2/20 (10.0%) 1/20 (5.0%) 3/20 (15.0%) 1/20 (5.0%)
Pancreatic trauma	6/20 (30.0%)
(**b**)	
Number of ERCP procedures	62
Chronic pancreatitis Acute pancreatitis Main pancreatic duct rupture Recurrent acute pancreatitis Pancreas divisum	34/62 (54.8%) 6/62 (9.6%) 10/62 (16.1%) 11/62 (17.7%) 25/62 (40.3%)
Concomitant EUS Concomitant single operator pancreatoscopy	8/62 (12.9%) 2/62 (3.2%)

Abbreviations: EUS, endoscopic ultrasound.

**Table 2:** Therapeutic interventions and findings.

Minor papilla cannulation	10/62 (16.1%)
Pancreatic sphincterotomy	15/62 (24.2%)
Balloon dilation	15/62 (24.1%)
Catheter dilation	2/62 (3.2%)
Dilated MPD	48/62 (77.4%)
MPD diameter, mm	4–11
MPD stricture	36/62 (58.0%)
MPD stones	22/62 (35.4%)
MPD plugs	26/62 (41.9%)
Pseudocyst	25/62 (43.1%)
Pancreatic stenting	54/62 (87.0%)
Re-stenting	38/62 (61.2%)
ERCP performed only for stent removal	8/62 (12.9%)

Abbreviations: MPD, main pancreatic duct; ERCP, endoscopic retrograde cholangiopancreatography.

**Table 3:** Short- and Long-Term Outcomes of Endoscopic Procedures.

Short-term:	
Technical success	57/62 (91.9%)
Post-ERCP pancreatitis	2/62 (3.2%)
Bleeding	1/62 (1.6%)
Perforation	0/62 (0%)
ERCP-related death	0/62 (0%)
Long-term:	
Recurrent AP requiring surgery	1 (5.0%)
Chronic pain requiring surgery	1 (5.0%)
Surgery due to MPD rupture	2 (10.0%)

Abbreviations: ERCP, endoscopic retrograde cholangiopancreatography; AP, acute pancreatitis; MPD, main pancreatic duct.

**Table 4:** Demographic overview, short- and long-term outcomes at patient level.

Patient	Sex M/F	Age at the First ERCP	Number of Procedures	Diagnosis	Post Operative Complications	Long Term Outcome
1	M	12	4	CP	1 mild post-ERCP AP	Pain free
2	M	11	1	CP	None	Pain free
3	F	11	1	CP	None	Pain free
4	F	2	1	AP	None	Pain free
5	F	8	5	CP	None	Chronic pain Surgery
6	F	10	1	Insulinoma	None	Pain free
7	F	3	8	RAP	None	Pain improvement. Surgery at age 7
8	M	5	2	AP	None	Pain free
9	M	10	1	MPD rupture	None	Pain free
10	F	10	1	MPD rupture	None	Surgery
11	M	9	1	MPD rupture	None	Surgery
12	M	4	10	CP	None	Pain improvement
13	M	7	3	MPD rupture	Bleeding	Pain free
14	F	5	2	AP	None	Pain free
15	M	12	2	MPD rupture	None	Pain free
16	M	10	1	RAP/CP	None	Died during follow-up because of the complications of the background genetic disease
17	M	9	2	RAP	1 mild post-ERCP AP	Pain free
18	F	2	8	CP	None	Pain free
19	F	11	5	CP	None	Pain free
20	M	11	3	MPD rupture	None	Pain free

Abbreviations: ERCP, endoscopic retrograde cholangiopancreatography; AP, acute pancreatitis; RAP, recurrent acute pancreatitis; CP, chroinic pancreatitis; MPD, main pancreatic duct.

## Data Availability

The data presented in this study are available on request from the corresponding author.
